# Statistical modeling for identifying chikungunya high-risk areas of two large-scale outbreaks in Thailand's southernmost provinces

**DOI:** 10.1038/s41598-023-45307-9

**Published:** 2023-11-03

**Authors:** Lumpoo Ammatawiyanon, Phattrawan Tongkumchum, Don McNeil, Apiradee Lim

**Affiliations:** Department of Mathematics and Computer Science, Faculty of Science and Technology, Prince of Songkla University, Pattani Campus, Pattani, 94000 Thailand

**Keywords:** Diseases, Risk factors

## Abstract

Chikungunya fever (CHIKF) has re-emerged in the southernmost Thailand and presents a significant threat to public health. The problem areas can be identified using appropriate statistical models. This study aimed to determine the geographic epidemic patterns and high-risk locations. Data on CHIKF’s case characteristics, including age, gender, and residence sub-district, were obtained from the Office of Disease Prevention and Control of Thailand from 2008 to 2020. A logistic model was applied to detect illness occurrences. After removing records with no cases, a log-linear regression model was used to determine the incidence rate. The results revealed that two large-scale infections occurred in the southernmost provinces of Thailand between 2008 and 2010, and again between 2018 and 2020, indicating a 10-year epidemic cycle. The CHIKF occurrence in the first and second outbreaks was 28.4% and 15.5%, respectively. In both outbreaks of occurrence CHIKF, adolescents and working-age groups were the most infected groups but the high incidence rate of CHIKF was elderly groups. The first outbreak had a high occurrence and incidence rate in 39 sub-districts, the majority of which were in Narathiwat province, whilst the second outbreak was identified in 15 sub-districts, the majority of which were in Pattani province. In conclusion, the CHIKF outbreak areas can be identified and addressed by combining logistic and log-linear models in a two-step process. The findings of this study can serve as a guide for developing a surveillance strategy or an earlier plan to manage or prevent the CHIKF outbreak.

## Introduction

Chikungunya, commonly known as chikungunya fever (CHIKF), is a serious public health problem caused by the chikungunya virus (CHIKV), which was initially found in Tanzania^1^ has infiltrated the worldwide health system over the last seven decades^2^. Its name is derived from a Makonde phrase that describes the bent position of people suffering from severe arthralgia caused by the infection. CHIKF is a mosquito-borne arbovirus spread by *Aedes aegypti* and, since 2006, also by *Aedes albopictus* mosquitoes^3^. Over the last 30 years, this new vector has invaded almost every country worldwide^4^. The infection usually manifests as fever and acute incapacitating joint pain, which can progress to prolonged and painful arthritis that can last months or years^5–7^. Once a person has recovered, they are likely to be immune to the disease^8^.

In Thailand, the first case of CHIKF infection were reported in Bangkok in 1958^9^ as were the first cases in Asia^10^. Subsequently, sporadic cases of CHIKF have been detected in many locations across Thailand from 1976 to 1995^11^. It is unclear how long the transmission persisted at low levels in this period, but there were relatively few cases reported between 1995 and 2008^12^, possibly because the signs and symptoms of CHIKF are similar to those of dengue^13^. As a result, incidents may often be documented under either diagnosis. Also, the Thailand Epidemiological Surveillance Report did not include CHIKF until 2008, when it began reporting^14^. In August 2008, the disease was first found in Yi-ngo district, Narathiwat province^15^. Then, the disease quickly spread across Thailand^16^. Before Thailand’s large-scale outbreak in 2008, Malaysia had a CHIKF outbreak in 2006 in the state capital of Perak^17,18^ which is near the Thai border. According to the Bureau of Epidemiology, Thailand’s reported CHIKF cases were 2494 in 8 provinces, 52,057 in 58 provinces, and 1565 in 32 provinces in 2008, 2009, and 2010, respectively, and affected mainly the southern region of the country. Following a drop in CHIKF infections in 2010, a second large-scale outbreak reappeared in 2018–2020^19^. The outbreak started in Satun province, where a predominantly rural region borders northern Malaysia^20^. Unfortunately, there is no specific antiviral treatment and no effective vaccinations, therefore treatment is only supportive^21^.

CHIKV has the potential to cause large outbreaks with high attack rates, potentially overburdening the healthcare system by affecting one-third to three-quarters of the population in areas where the virus is circulating. Both large-scale outbreaks have the potential to spread the disease across the country and worldwide to previously non-endemic areas^22,23^ when the locations provide suitable environmental conditions for autochthonous CHIKV transmission^24^. Both the *Aedes albopictus* and *Aedes aegypti* species of mosquito can be carriers of this virus^25^. *Aedes albopictus* predominates in rural areas and *Aedes aegypti* in urban areas. Therefore, cases are common in both urbanized^26^ and rural areas^11^. The mosquitoes that carry the disease often venture out to feed during the daytime, meaning that all age groups who spend time outside the home are most at risk of contracting the disease. The majority of CHIKF cases were found in people over the age of ten^19^.

The majority of people in the southernmost provinces are Thai Muslims who have strong connections with the Malay community and frequently cross the border to work in Malaysia^27^, which has chikungunya-endemic areas^18^. A lack of access to chikungunya prevention, diagnostic and treatment options, and inadequate monitoring measures could all contribute to chikungunya outbreaks in border regions^15^. As a result, CHIKF spread at an alarming rate in the region^19,28^. There has been no research in this area on the distribution of CHIKF in the two large outbreaks described. In some predictor categories, analysis of CHIKF data frequently encounters the issue of zero cases. Overdispersion may result from the high fraction of zeros, indicating that the data and the assumed distribution disagree. We hypothesized that the high-risk locations of the two outbreaks could be identified if the data were separately analyzed for each outbreak using the two-step process. The aim of our study was to identify high-risk sub-districts and gender–age patterns based on modeling of the 2008–2010 and 2018–2020 ddata (Supplementary Information).

## Methods

**Figure 1:**
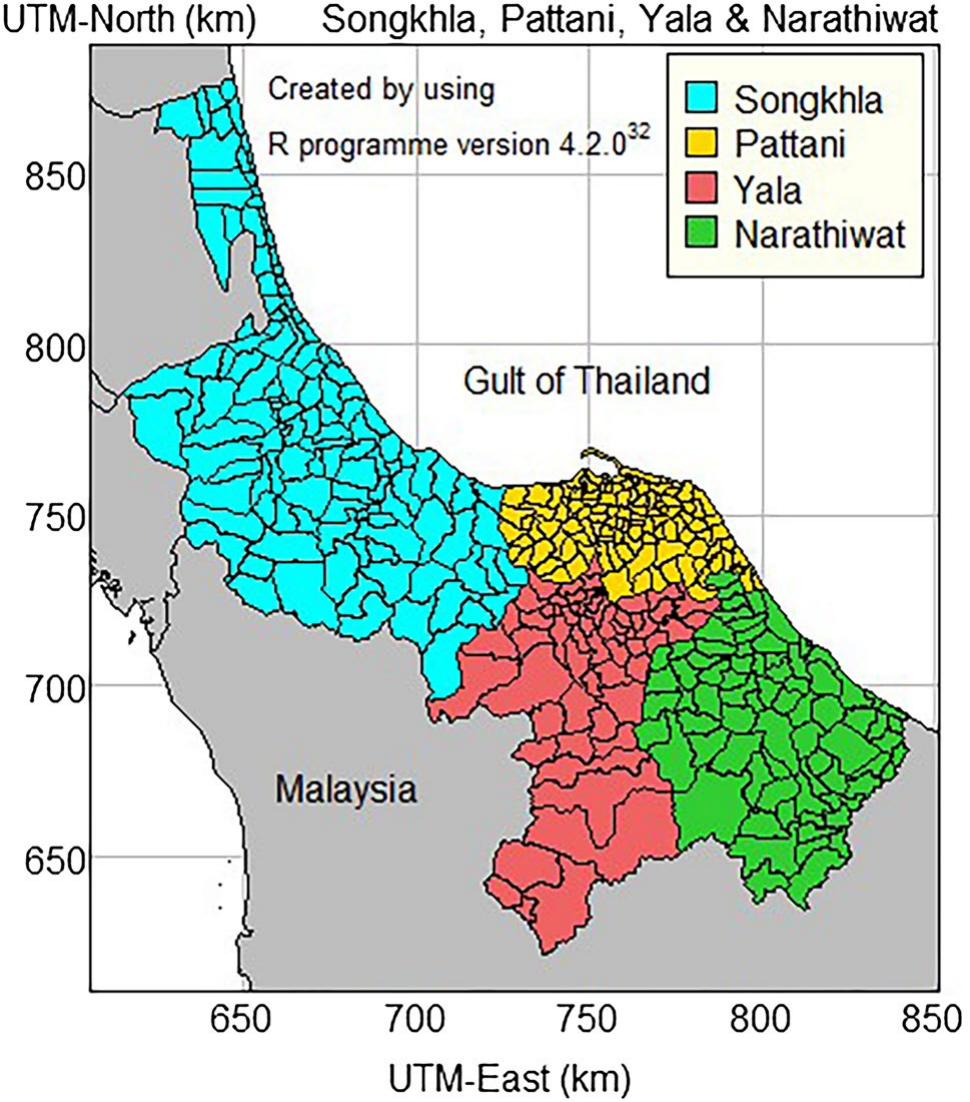
Sub-district-level map of the four southernmost provinces in Thailand.

The study area comprised Thailand's four southernmost provinces (Songkhla, Pattani, Yala, and Narathiwat), of which Songkhla, Yala, and Narathiwat all share parts of their borders with Malaysia. There are 377 sub-districts in 49 administrative districts in these four provinces (Fig. 1). In 2020, the total population of these four provinces was over 3.4 million^29^.

A total of 35,059 hospitalized CHIKF patients’ records from 2008 to 2020, which included age, gender, year, sub-district of residence, and province, were obtained from the Office of Disease Prevention and Control, Ministry of Public Health. The CHIKF cases in these data were diagnosed based on clinical symptoms, which were all new cases, as each CHIKF case was confirmed by the epidemiologists in order to control the disease. Population counts from 2008 to 2020, separated by age group, gender, and sub-district of each province, were obtained from the Department of Provincial Administration, Ministry of Interior, Thailand. The data for the two outbreaks that occurred between 2008 and 2010 and between 2018 and 2020 were chosen and separately analyzed.

Gender was classified as male or female. Age was divided into 8 groups with a 10-year interval:

0–9, 10–19, 20–29, 30–39, 40–49, 50–59, 60–69, and 70 and older. Gender and age group were combined to form a 16-category variable termed “gender–age group”. The data structure for statistical analysis for each outbreak thus comprised 18,096 (377 sub-districts × 3 years × 16 levels of gender–age group) records.

CHIKF incidence rates per 1000 population were calculated by dividing the number of CHIKF cases by the population of each gender, age group, sub-district, and year and then multiplying by 1000. The incidence rate for each record was used to define occurrence. If at least one CHIKF case was found in any records, the CHIKF occurrence code was coded as 1; otherwise, it was coded as 0.

The data analysis comprised two stages with the CHIKF occurrence and incidence rates being outcome variables. The CHIKF occurrence is a binary variable, whereas the incidence is a continuous variable. A two-step approach comprised of logistic model for occurrence and linear model for incidence rate was used^30^. These two models included sub-district, year, gender–age group as determinants. No-case records were excluded prior to the linear model analysis.

Logistic regression was applied to model the relationship between the occurrence outcome and the determinants. The population was categorized into four groups: less than 400, 400–599, 600–799, and 800 or more, and we utilized this as another predictor for occurrence outcome. The model was fitted using Eq. (1).

1 $$ln\left(\frac{{p}_{ijkl}}{1-{p}_{ijkl}}\right)=\mu +{\alpha }_{i}+{\beta }_{j}+{\delta }_{k}+{\gamma }_{l}$$

In this model, ${p}_{ijkl}$ denotes the probability of CHIKF occurrence in a combination of determinant factor levels and $\mu$ is constant. The terms ${\alpha }_{i}$, ${\beta }_{j}$, ${\delta }_{k}$ and ${\gamma }_{l}$ represent the effects of age-gender, year, sub-district, and population group.

The incidence rates of CHIKF had positively skewed distributions and were logarithmically transformed. The log-linear model of incidence rate is as follows:

2 $${\text{ln}}\left(1000 \times \frac{{n}_{ijk}}{{P}_{i}}\right)={y}_{ijk}=\mu +{\alpha }_{i}+{\beta }_{j}+{\delta }_{k}$$

In this model, ${n}_{ijk}$ represents the number of CHIKF cases recorded in sub-district $i$ and gender–age group $j$ of year $k$. ${P}_{i}$ represents the population of sub-district $i$.

Instead of treatment contrasts, the logistic and linear models were fitted using “sum contrasts”^31^. For each determinant level in the model, the 95% confidence intervals for occurrence and incidence rate were estimated. Based on these confidence intervals, sub-district thematic maps were constructed. The logistic regression model was assessed using the area under the receiver operating characteristic (ROC) curves (AUC), while the linear model was assessed using a quantile–quantile (Q–Q) plot of studentized residuals. All statistical analyses, graphical displays, and map creations were performed using R version 4.2.0^32^.

All methods in this study were carried out in accordance with relevant guidelines and regulations. This study and the requirement for informed consent have been approved and waived by the Human Research Ethics Committee of Prince of Songkla University, Pattani Campus, under approval number psu.pn 1-007/63.

## Results

**Figure 2:**
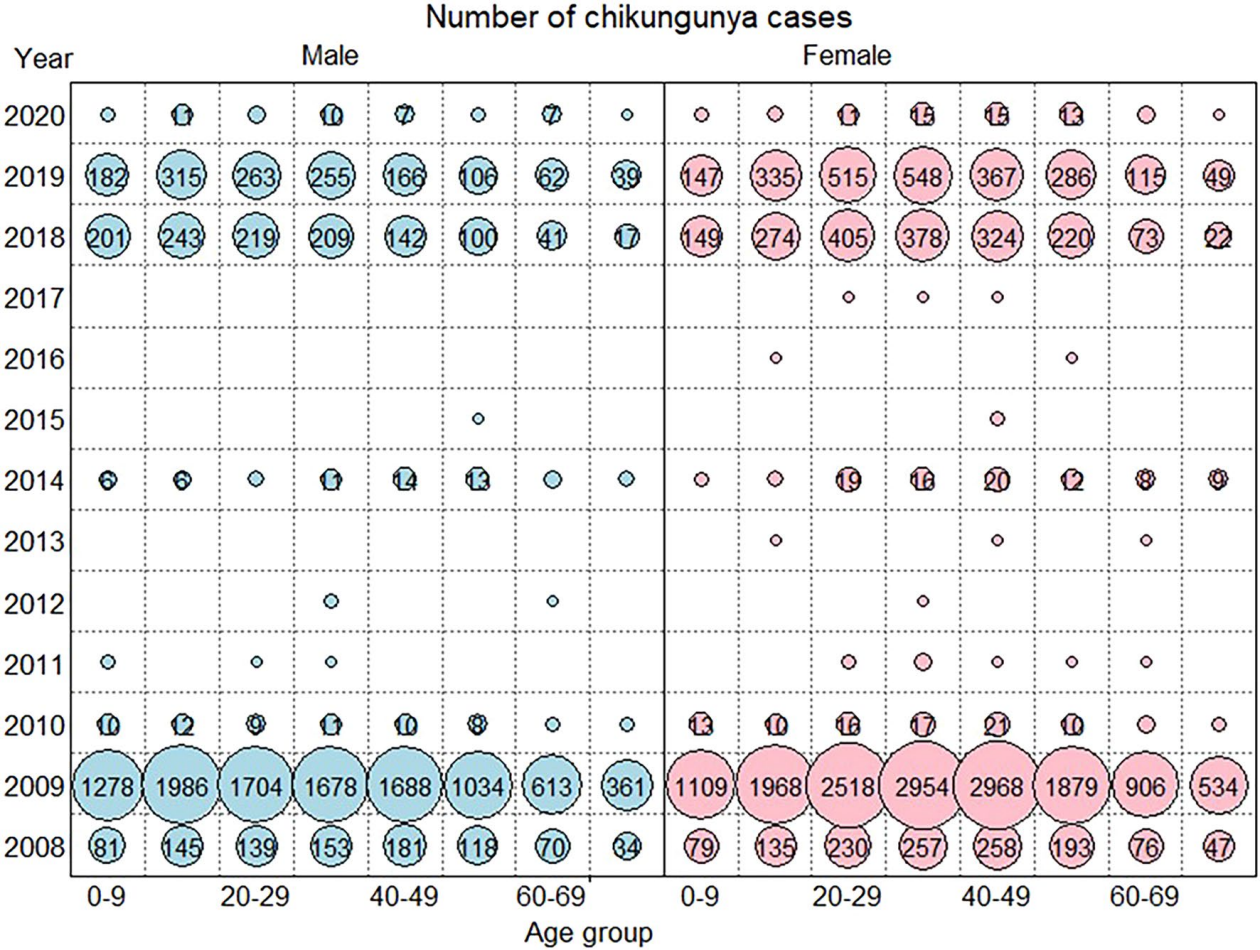
The numbers of CHIKF cases cross-classified by gender–age group and year.

In Thailand’s four southernmost provinces, 35,059 CHIKF infections were reported between 2008 and 2020. Figure 2 shows the numbers of CHIKF cases cross-classified by gender–age group and year. The bubble plot reveals that large-scale CHIKF case epidemics occurred in 2008–2010, and history repeated itself 10 years later with another large-scale outbreak in 2018–2020. It revealed a 10-year disease cycle that was observed across all age groups.

**Figure 3:**
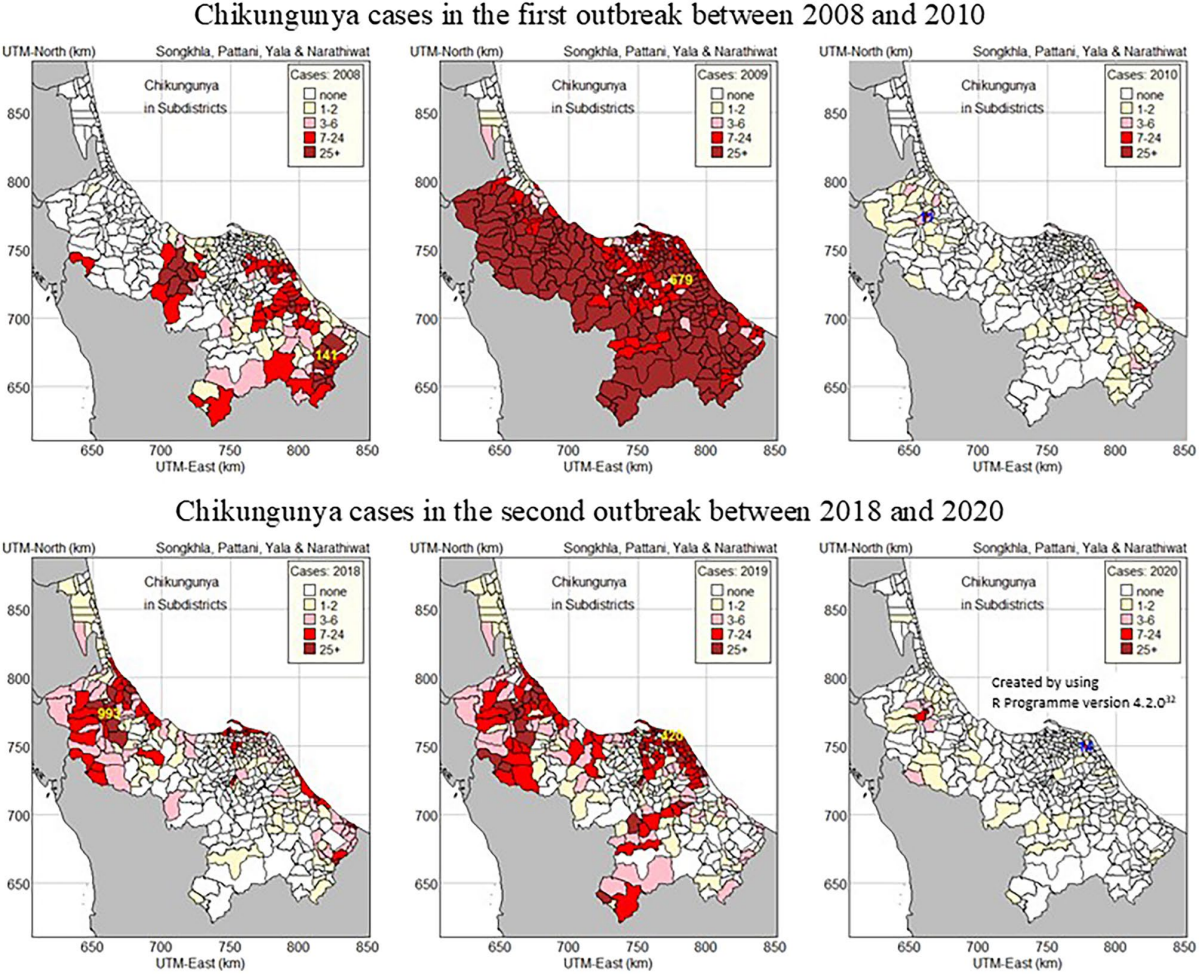
The number of hospitalized patients for CHIKF from 2008 to 2010 and 2018 to 2020 by sub-district.

The top row of Fig. 3 shows a thematic map for each year of the first large-scale outbreak, showing the total number of CHIKF hospitalized patients for each sub-district in the southernmost provinces from 2008 to 2010. In 2008, the total number of hospitalized patients ranged from 0 to 141. The outbreak manifested primarily along country and province borders. Within the country, it occurred along the borders of Songkhla and Yala provinces, Pattani and Yala, Pattani and Narathiwat, and Narathiwat and Yala. It occurred along the international border between Narathiwat and Malaysia. In 2009, CHIKF had spread to the four southernmost provinces of Thailand, with the exception of the northern part of Songkhla province, where the number of cases was relatively low. The Yi-ngo sub-district in Narathiwat province had the greatest number of CHIKF cases in 2009, with 679 cases. The number of hospitalized patients decreased in all sub-districts in 2010.

The bottom row of Fig. 3 shows a thematic map for each year of the second large-scale outbreak, showing the total number of CHIKF hospitalized patients for each sub-district in the southernmost provinces from 2018 to 2020. In 2018, the outbreak began at the border between Songkhla province and Satun province, as well as the border between Songkhla province and Malaysia. The majority of CHIKF cases in 2018 were reported in Hat Yai, which is located in central Songkhla province (993 cases). CHIKF extended from Songkhla to Pattani and Yala in 2019, whereas Narathiwat had fewer cases. In 2020, the outbreak declined in all sub-districts.

Table 1 summarizes the characteristics of the CHIKF occurrence and incidence rates in 2008–2010 and 2018–2020. The first outbreak of occurrence (2008–2010) peaked in 2009 corresponding to incidence rates. The highest occurrence rates were in males aged 20–29 and females aged 30–39 years, whereas the highest incidence rates were in those aged 60–69 years for both males and females. The highest occurrence and incidence rate were in Narathiwat province.

**Table 1:** Occurrence and incidence rate of CHIKF by demographic factors in two outbreaks.

Determinates	First outbreak		Second outbreak	
	Occurrence	Incidence	Occurrence	Incidence
Year				
2008	15.49	6.57	–	–
2009	67.09	16.63	–	–
2010	2.70	1.66	–	–
2018	–	–	16.88	2.84
2019	–	–	27.54	4.64
2020	–	–	2.015	2.01
Gender–age group				
Male				
0–9 years	26.04	6.91	12.38	2.93
10–19 years	31.00	9.66	17.70	3.50
20–29 years	33.14	8.82	17.81	3.02
30–39 years	31.49	10.75	17.60	2.79
40–49 years	31.20	13.70	13.90	2.98
50–59 years	26.24	16.32	9.99	3.64
60–69 years	19.34	22.51	8.69	5.97
70 + years	14.97	19.81	5.86	7.21
Female				
0–9 years	26.92	5.98	9.99	2.75
10–19 years	32.36	9.50	16.94	3.65
20–29 years	35.96	12.52	26.72	4.38
30–39 years	36.15	16.63	27.58	4.35
40–49 years	31.20	19.86	22.26	3.87
50–59 years	23.52	22.10	19.22	3.58
60–69 years	19.34	24.36	13.14	5.38
70 + years	26.92	16.65	7.93	4.32
Province				
Yala	22.92	10.19	9.45	2.47
Pattani	21.54	15.04	16.58	6.44
Narathiwat	42.15	15.26	10.20	2.06
Songkhla	28.89	14.51	18.49	2.61

The second outbreak (2018–2020) peaked in 2019 with comparable incidence rates. Males aged 10–39 and females aged 30–39 had the highest occurrence rate, while males over 70 and females 60–69 had the highest incidence rate. The highest occurrence was in Songkhla province, while the incidence rates were highest in Pattani province.

**Figure 4:**
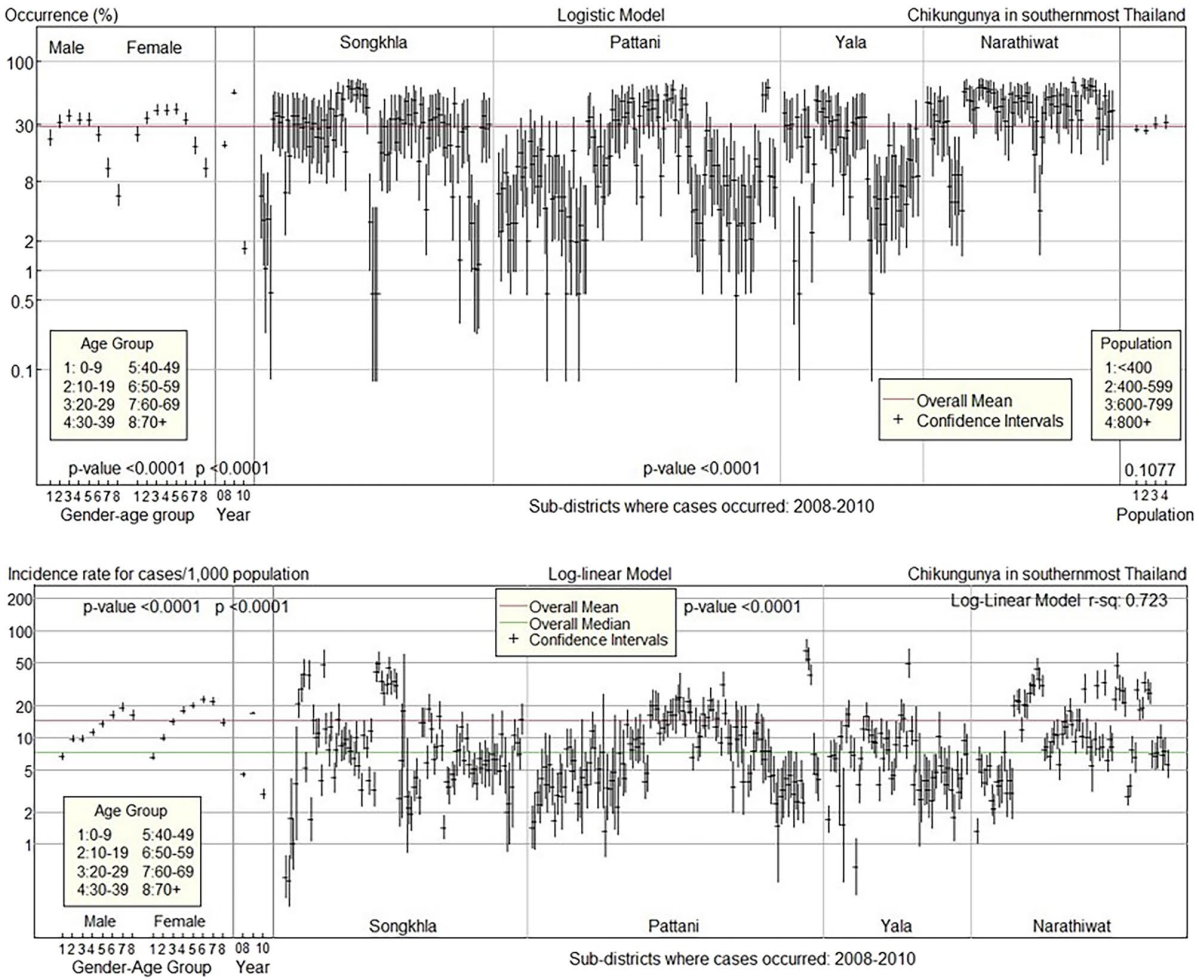
Confidence interval plots for CHIKF occurrence and incidence rate in 2008–2010.

The top panel of Fig. 4 illustrates predicted occurrences and confidence intervals in the first pandemic (2008–2010) using gender–age group, year, sub-district, and demographic group as predictors. The overall occurrence rate was 28.4%. Male and female occurrence patterns were similar. The occurrence rate increased with age, with males substantially higher than the overall mean at ages 20–49 and females at ages 10–59, before falling significantly below the overall mean at ages 60 and older for both sexes. Most sub-districts in Songkhla and Narathiwat provinces had above-mean occurrences.

The bottom panel of Fig. 4 shows the log-linear model’s confidence intervals for the CHIKF incidence rate in 2008–2010 for each predictor. The overall median incidence rate was 7.3 cases per 1000 population, whereas the overall mean was 14.3 cases per 1000 population. The results were then compared to the median incidence rate due to the wide variation in incidence rates. The incidence rate patterns in males and females are similar. The incidence rates increased with age, peaking in the older group and then dropping after the age of 70 for both genders. The provinces of Songkhla and Narathiwat had the greatest number of sub-districts with above-median incidence rates.

**Figure 5:**
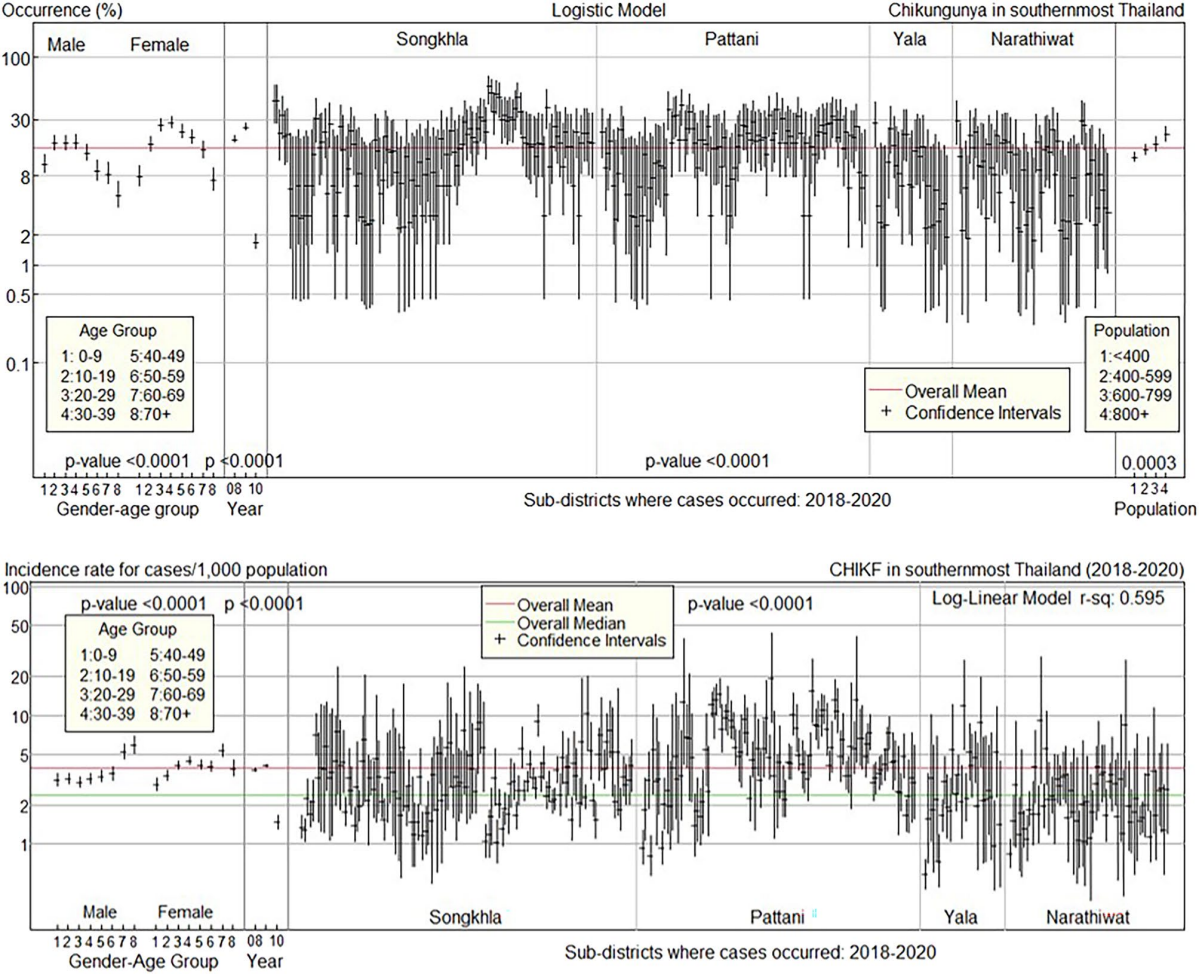
Confidence interval plots for CHIKF occurrence and incidence rate in 2018–2020.

The top panel of Fig. 5 depicts the predicted occurrences with gender–age group, year, sub-district, and population group as predictors, as well as their confidence intervals, during the second outbreak (2018–2020). The overall occurrence rate was 15.5%. The occurrence patterns of the second outbreak in gender and age categories are comparable to those of the prior outbreak. The occurrence among females aged 20–59 was significantly higher than the overall mean, whereas the occurrence among males aged 0–9 and those aged 50 and older, as well as females aged 0–9 and those aged 70 and older, was significantly lower than the overall mean. Most sub-districts in Songkhla and Pattani provinces reported above-mean occurrences.

The confidence intervals for the CHIKF incidence rate in 2018–2020 for each predictor are shown in the bottom panel of Fig. 5. The overall median incidence rate was 2.4 cases per 1000 population, whereas the overall mean was 3.9 cases per 1000 population. Males and females have differing incidence rates. Males’ incidence rates grew with age, whereas females’ incidence rates peaked at 60–69 years and fell at 70 years and older. Most sub-districts in Songkhla and Pattani provinces showed an above-average incidence rate.

The logistic model for the first outbreak had a predictive accuracy of 88.1% and an AUC of 90.2%, whereas the logistic model for the second outbreak had a predictive accuracy of 86.3% and an AUC of 78.6%, indicating a good fit. A linear model for predicting the transformed CHIKF incidence rate by logarithm of both outbreaks produced good fits as the residuals in the quantile–quantile (Q–Q) plot of studentized residuals tended to follow a diagonal line with R^2^ values of 72.3% and 59.5%, respectively.

Thematic maps were used to categorize sub-districts into three groups based on whether the confidence intervals of CHIKF occurrence were entirely above, around, or below the overall mean. The overall median was used to categorize sub-districts into three groups using the same criteria as the incidence rate.

**Figure 6:**
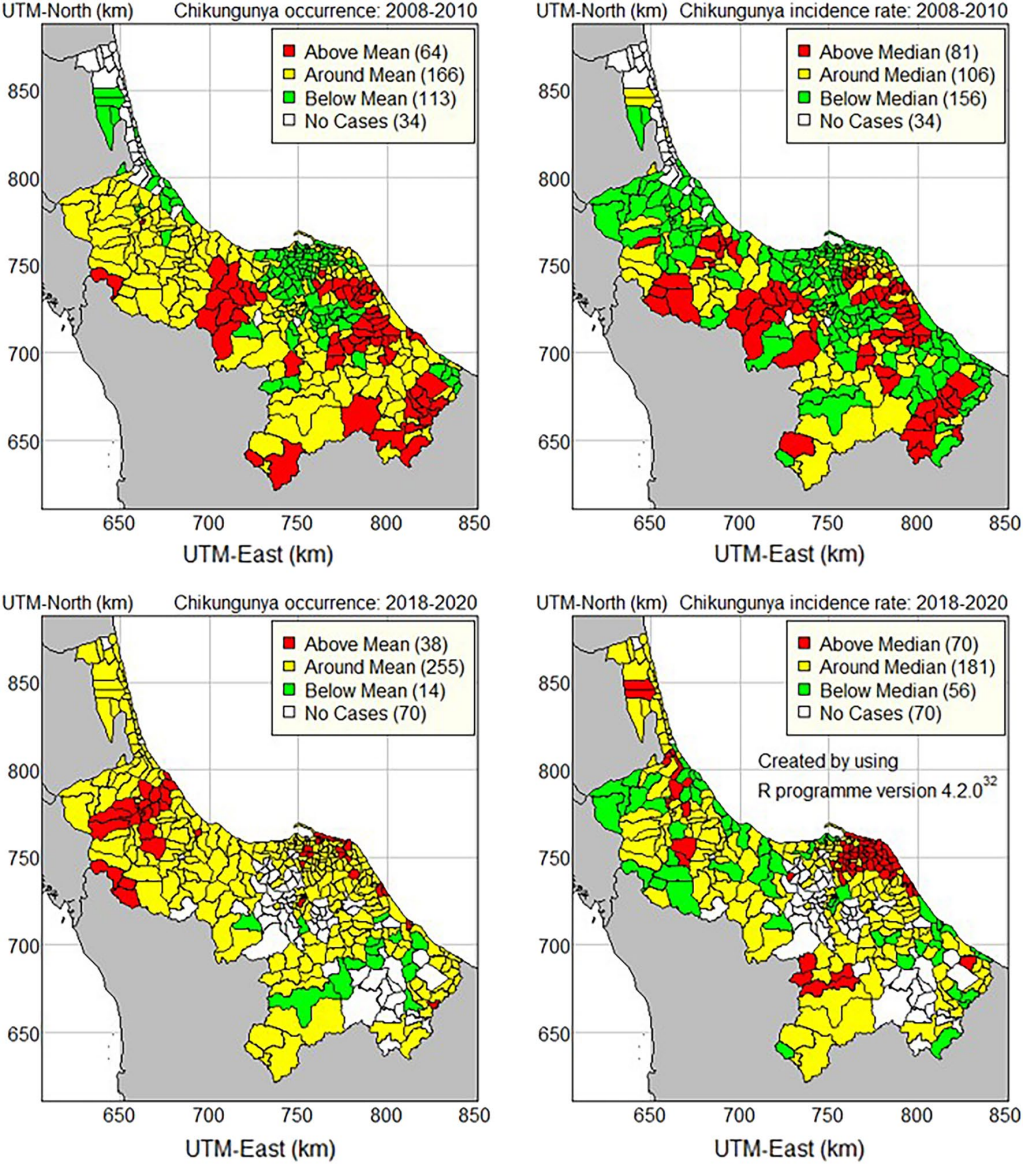
Thematic maps of CHIKF occurrence and incidence rate in 2008–2010 and 2018–2020 in the southernmost provinces of Thailand.

The thematic maps are shown in the top panel of Fig. 6, with the occurrence of CHIKF in the first outbreak (2008–2010) in the top-left panel and the CHIKF incidence rate in the top-right panel. The occurrence of the CHIKF outbreak was greater than the overall mean of 64 sub-districts, while its incidence rate was greater than the overall median of 81 sub-districts. Narathiwat province had the most sub-districts with an above-average occurrence during the first outbreak, with 35, followed by Pattani with 14, Songkhla with 12, and Yala with 3. Similarly, Narathiwat province had the most sub-districts with an above-average incidence rate, with 30, followed by Pattani province with 25, Songkhla province with 21, and Yala province with 5, respectively.

The bottom of Fig. 6 shows the thematic maps with the occurrence of CHIKF in the second outbreak (2018–2020) in the bottom-left panel, while the incidence rate of CHIKF is shown in the bottom-right panel. The occurrence of the CHIKF outbreak was greater than the overall mean in 38 sub-districts, whereas the incidence rate was greater than the overall median in 70 sub-districts. During the second outbreak, Songkhla province had the highest number of sub-districts with an above-mean occurrence, with 21 sub-districts, followed by Pattani with 14, Narathiwat with 2, and Yala with 1. Pattani province had the most sub-districts with an incidence rate above the median (51 sub-districts), followed by Songkhla with 14, Yala with 4, and Narathiwat with 1 sub-district.

**Figure 7:**
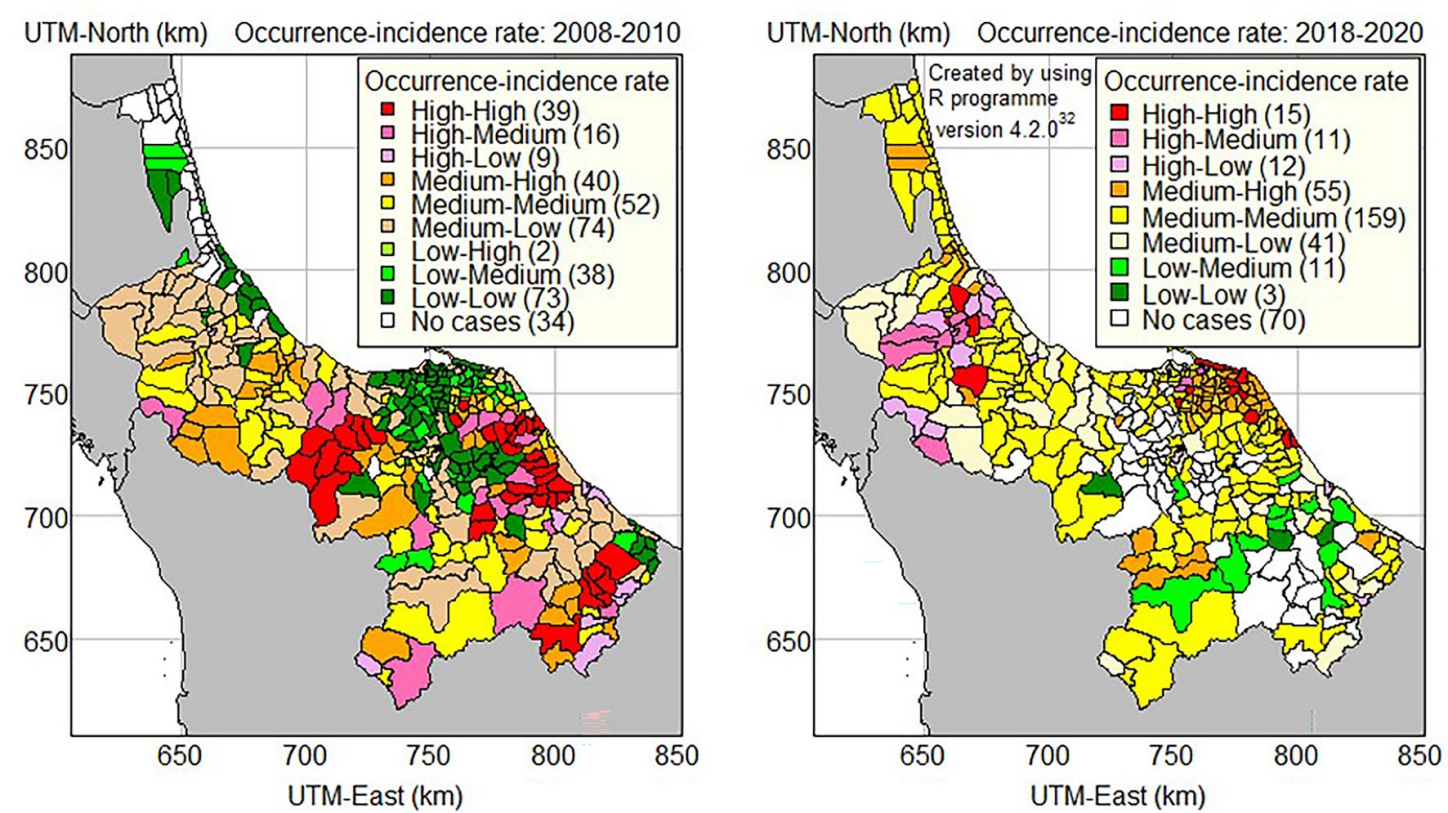
Occurrence-incidence rate map of CHIKF in 2008–2010 and 2018–2020 in the southernmost provinces of Thailand.

Figure 7 depicts a thematic map of all combinations of occurrence and incidence rate levels. The first outbreak (2008–2010) is represented in the left panel, whereas the second outbreak is shown in the right panel. Chikungunya occurrence and incidence rates were recorded for 39 and 15 sub-districts, respectively, in the high-high group. In the first outbreak, the majority of the high-high occurrence and incidence rate sub-districts were identified in Narathiwat, Pattani, and Songkhla provinces. In the first outbreak, there were 32, and 2 sub-districts in Songkhla and Yala provinces with free CHIKF infections, respectively; whereas in the second outbreak, there were 30, 19, 15, and 6 sub-districts in Yala, Narathiwat, Pattani and Songkhla provinces with free CHIKF infections, respectively.

## Discussion

For this study, the incidence rates for two large-scale outbreaks in Thailand's southernmost provinces were divided into occurrences and incidence rates without zeros. A logistic model for occurrence and a log-linear regression model for CHKIF incidence rates were separately fitted, and the results were merged. This two-stage analysis has previously been described^30^. This method can be applied to rare diseases with data that contains a large proportion of zeros. The approach developed in our study was used to identify areas with high CHIKF occurrences and incidence rates among susceptible persons, allowing public health officials to prevent impending epidemics.

For both genders, the first outbreak occurrence increased until the ages of 20–29, then decreased after age 50, while the incidence rate increased with age and decreased after age 70. The occurrence of the second outbreak in females increased until the age of 30–39, then decreased in both genders after the age of 40 and older. The incidence rate of the second outbreak increased with age, except for females, who declined around age 70 and older. The CHIKF epidemic had a 10-year cycle, with peaks in 2009 and 2019. The majority of sub-districts in Narathiwat, Songkhla, and Pattani provinces had above-average occurrence and incidence rates.

The occurrence of the first outbreak increased in early adulthood and decreased with age. The result is consistent with a study conducted in India, which revealed that the majority of cases (56%) were found among those aged 15–44^33^. The majority of the young adults in this area work on rubber plantations, where they spend the majority of their time outside, increasing their risk of getting CHIKF^34,35^. On the other hand, the incidence rate of first outbreak increased with age and decreased after age 70. Our findings are consistent with the Bureau of Epidemiology's official records, which revealed that the 45–64 age group had the highest incidence in 2008^15^. This may be due to the fact that older age is obviously the risk group for the development of arthralgia in infected patients^7^ who are unable to receive adequate treatment at home and have to be transferred to a hospital. Furthermore, the elderly has a lower immune response than younger people, making them more prone to illnesses^36^. Older persons may have underlying health issues, such as diabetes or cardiovascular disease^37^, that weaken their immune system or make them more vulnerable to chikungunya consequences. Once an outbreak of CHIKF occurs, this age group could occur a high number of infections.

The second outbreak occurrence peaked in adolescence and early adult groups and decrease at aged 40 and older. This may be due to the fact that these populations have not been afflicted with the disease within the last decade, and therefore lack protective antibodies. It has been discovered that CHIKV shows long-term persistence of neutralizing antibodies in human populations in northern and eastern Thailand, 19 years after infection^38^. As a result, the majority of infection cases occurred in adolescence and early adult.

In the first round, the incidence of CHIKF outbreaks increased with age and decreased after age 70, whereas in the second round, the incidence increased with age for males but decreased at age 70 for females. This finding is consistent with official reports from the Bureau of Epidemiology, which indicated that the age group between 45 and 64 had the highest incidence of the 2008^15^. If only prevalence is considered, CHIKF is prevalent among young adults, whereas its incidence increases with age. This group may have participated in fewer outdoor activities and had less contact with the risk group, whose rate decreased in this age group.

Our findings revealed a 10-year cycle of CHIKF outbreaks. This finding affirms the findings of Chusri et al., who found that large-scale CHIKF outbreaks occurred between 2008 and 2010^39^. A second large-scale outbreak was reported between 2018 and 2020, 10 years after the first outbreak^40^. This indicates a 10-year disease cycle, which was also identified in an Italian study^41^. According to the World Health Organization, CHIKF epidemics follow a cyclical pattern with an interepidemic period of 4–8 years, and occasionally up to 20 years^42^. A smaller number of people were affected by the second outbreak in 2018–2020 compared to the first outbreak in 2008–2010. Prior to 2008, the only known strains of CHIKF circulating in Thailand belonged to the Asian lineage^9^. The 2008 outbreak was caused by the introduction of East Central and South African lineages. CHIKF isolates from the 2008 outbreak carried the A226V point mutation, which has been shown to increase vector specificity for *Aedes albopictus*^43^. The second outbreak, on the other hand, had a different genotype than the first^44^, resulting in a dramatically increased incidence of CHIKF in the southernmost provinces.

This study has few limitations. Firstly, the analysis results of this study may be influenced by the fact that not all cases of CHIKF in the data were confirmed by laboratory tests. However, CHIKF cases were laboratory confirmed if physicians were unsure, and laboratory confirmation was mostly done when the CHIKF epidemic began, such as in 2008–2010^39,45^ and 2018–2020^19^. Secondly, patients with fewer symptoms may not seek hospital care, and some patients in rural areas may not seek health care, which could lead to underreporting in the data used for this study. Thirdly, environmental factors including temperature, humidity, land use, and land cover, as well as mosquito data including the container index, house index, and Breteau index, which were reported to have had a significant impact on the CHIKF outbreak, were not considered in this study.

## Conclusion

Two large-scale CHIKF outbreaks occurred between 2008 and 2010 and between 2018 and 2020, following a 10-year cycle of outbreaks. The occurrence of CHIKF decreased after 50 years of age and older in the first outbreak and 10 years earlier in the second outbreak, at age 40 and older. Except for those aged 70 and older in the first outbreak and females in the second outbreak, the incidence of CHIKF outbreaks increased with age. Occurrence and incidence for the regions affected by the first outbreak tended to disappear or have fewer problems in the second outbreak, suggesting that a person is immune to the illness once they have been infected by CHIKF. Identifying issue regions can be approached through a combination of occurrence and incidence rate. Therefore, problem areas can be assigned appropriate measures and policies. Further study is needed on the duration of immunity and the CHIKF epidemic in non-infected areas.

### Supplementary Information

Supplementary Information.

## Data Availability

The datasets used and/or analyzed during the current study are available from the corresponding author on reasonable request.
